# Genetic control of inflorescence architecture during rice domestication

**DOI:** 10.1038/ncomms3200

**Published:** 2013-07-25

**Authors:** Zuofeng Zhu, Lubin Tan, Yongcai Fu, Fengxia Liu, Hongwei Cai, Daoxin Xie, Feng Wu, Jianzhong Wu, Takashi Matsumoto, Chuanqing Sun

**Affiliations:** 1 State Key Laboratory of Plant Physiology and Biochemistry, National Center for Evaluation of Agricultural Wild Plants (Rice), China Agricultural University, Beijing 100193, China; 2 Tsinghua-Peking Center for Life Sciences, School of Life Sciences, Tsinghua University, Beijing 100084, China; 3 Key Laboratory of Plant Molecular Physiology, Institute of Botany, Chinese Academy of Sciences, Beijing 100093, China; 4 National Institute of Agrobiological Sciences, Tsukuba 305-8602, Japan

## Abstract

Inflorescence architecture is a key agronomical factor determining grain yield, and thus has been a major target of cereal crop domestication. Transition from a spread panicle typical of ancestral wild rice (*Oryza rufipogon* Griff.) to the compact panicle of present cultivars (*O. sativa* L.) was a crucial event in rice domestication. Here we show that the spread panicle architecture of wild rice is controlled by a dominant gene, Os*LG1,* a previously reported SBP-domain transcription factor that controls rice ligule development. Association analysis indicates that a single-nucleotide polymorphism-6 in the Os*LG1* regulatory region led to a compact panicle architecture in cultivars during rice domestication. We speculate that the *cis*-regulatory mutation can fine-tune the spatial expression of the target gene, and that selection of *cis*-regulatory mutations might be an efficient strategy for crop domestication.

## Abstract (web summary)

Transition from a spread panicle typical of ancestral wild rice to the compact panicle of present cultivars was a crucial event in rice domestication. Here the authors show that this panicle architecture is controlled by the transcription factor OsLG1 and that a mutation in its regulatory region led to the compact panicle phenotype.

## Graphical abstract

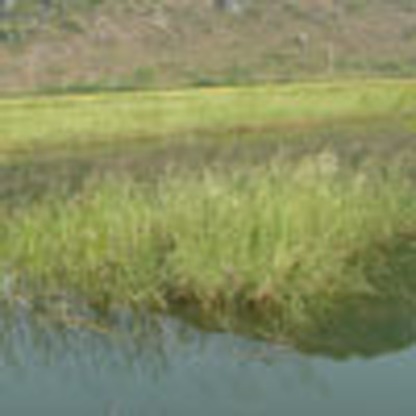

Inflorescence architecture is a key agronomical factor determining grain yield, and thus has been a major target of crop domestication and improvement^1^,^2^. Understanding the genetic basis of inflorescence architecture will contribute to not only elucidating the crop evolutionary mechanism but also improving crop grain yield.

Asian cultivated rice (*Oryza sativa* L.), one of the oldest domesticated crops, now feeds over one-third of the global population^3^. Morphological and physiological traits had been remarkably changed during the domestication of rice. Recently, evolutionary mechanisms and genetic factors controlling rice domestication-related traits, such as grain shattering, prostrate growth habit, pericarp colour and grain size have been characterized^4^,^5^,^6^,^7^,^8^,^9^,^10^,^11^,^12^. The transition in inflorescence architecture from a spread panicle to a compact panicle was another critical event for rice domestication. Common wild rice (*O. rufipogon* Griff.) displays typical architecture of a spread rice panicle (Fig. 1a), which may be adapted to its out-crossing pollination habit, contribute to seed dispersal at maturity and reduce the risk of pathogen infection^13^,^14^. These characteristics of wild rice are beneficial to maintain a high genetic diversity and permit adaptive changes to natural ecological environments. In contrast, cultivated rice has a compact panicle (Fig. 1b), which may be more amenable to high-density planting once rice plants grow erect, improve harvest efficiency, bear more seeds on the panicle branches and enhance photosynthetic efficiency of the lower leaves^2^,^15^. These characteristics of cultivated rice may increase grain-yield potential and meet the human’s needs of cultivation.

The spread panicle of *O. rufipogon* has been a classical trait for genetic studies of wild rice. In 1932, Mitra and Ganguli^16^ were the first to report that the spread panicle of wild rice was controlled by two complementary dominant genes This was confirmed in 1990 when a dominant single locus controlling panicle spread in wild rice was identified on chromosome 4 (ref. 17). However, until now the molecular basis for this key event in rice domestication has not been elucidated.

Here we characterize an introgression line that exhibits spread panicle phenotype and find that the spread panicle architecture of wild rice is controlled by a dominant gene, *OsLG1*, a previously reported SBP-domain transcription factor that controls rice ligule development^18^. Association analysis indicates that a single-nucleotide polymorphism (SNP)-6, residing in the *cis*-regulatory region 11 kb upstream of the translation start site, affects the *O*s*LG1* gene expression, which is associated with a significantly altered cell morphology at the panicle pulvinus, and consequently leads to a compact panicle architecture in domesticated rice cultivars.

## Results

### Characterization of the spread panicle introgression line YIL31

**Figure 1:**
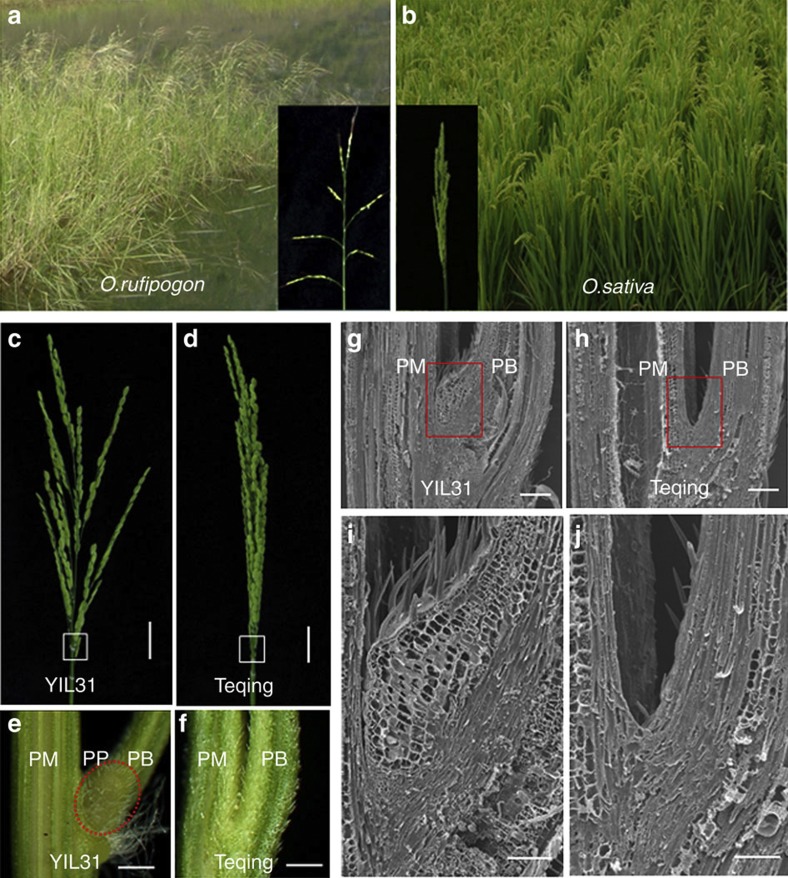
Phenotype characterization of wild rice and cultivated rice. (**a**,**b**) Phenotypes of *O. rufipogon* (**a**) and *O. sativa* (**b**). (**c**,**d**) Panicle of introgression lines YIL31 (**c**) and Teqing (**d**) at the heading stage. Scale bars, 3 cm. (**e**,**f**) Micrographs of the panicle internode of YIL31 (**e**) and Teqing (**f**) magnified from the boxed region in **c**,**d**, respectively. PM, panicle main axis; PB, panicle branch; PP, panicle pulvinus. Scale bars, 0.5 mm. (**g**–**j**) Scanning electron micrographs showing a longitudinal section of panicle internodes from introgression lines. YIL31 shows elongated parenchyma cells in the spread panicle (**g**,**i**) that are absent in the compact panicle phenotype of Teqing (**h**,**j**). Scale bars, 200 μm (**g**,**h**) and 100 μm (**i**,**j**).

In an attempt to identify the gene responsible for a spread panicle architecture, we constructed a set of introgression lines using an accession of Yuanjiang common wild rice (YJCWR, *O. rufipogon*) originating from Yuanjiang County, Yunnan Province, China, with a spread panicle architecture as a donor and an elite *indica* cultivar Teqing (*O. sativa*), displaying a compact panicle as the recipient^19^. One introgression line (YIL31) displaying a spread panicle, which harboured an YJCWR chromosome segment on the long arm of chromosome 4 (Supplementary Fig. S1), was selected for further study. Morphological analysis revealed that the panicle branch angle of YIL31 was much greater than that of Teqing (Fig. 1c,d), and a panicle pulvinus was present in YIL31 but absent in Teqing (Fig. 1e,f). Histological examination showed that the panicle pulvinus in YIL31 was composed of several layers of expanded parenchyma cells, which prevented the panicle branch from growing erect and resulted in a more angled growth pattern (Fig. 1g–j).

### Map-based cloning of *OsLG1*

**Figure 2:**
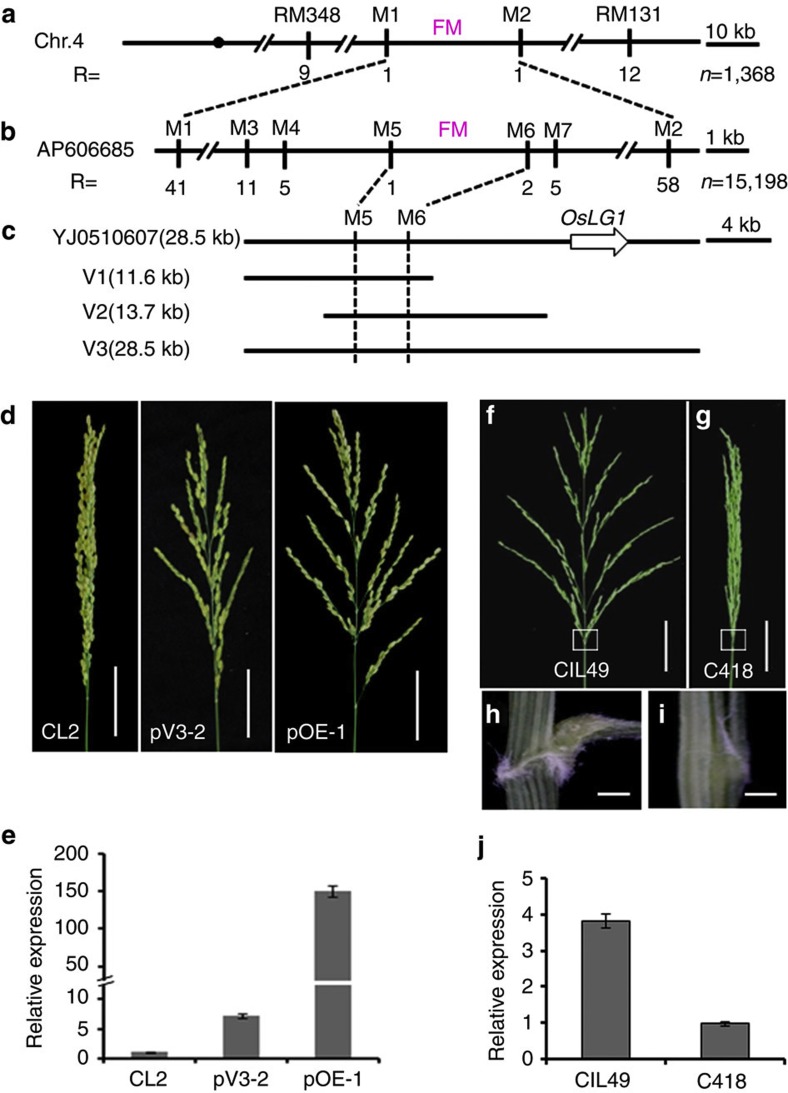
Map-based cloning of *OsLG1.* (**a**) The FM of the target gene was mapped between the markers M1 and M2 on the long arm of chromosome 4 by linkage analysis using 1,368 F_2_ individuals. (**b**) FM was finely mapped to a 3.3-kb region between markers M5 and M6 using 15,198 F_2_ individuals. (**c**) BAC clone YJ0510607 of YJCWR, which included *OsLG1* and the 3.3-kb-mapped segment as part of the upstream regulatory region. Genomic fragments V1 and V2 containing the 3.3-kb regulatory region; V3 included both *OsLG1* and the 3.3-kb regulatory region. (**d**) Complementation phenotypes of *japonica* cultivar Zhonghua 17: CL2, non-complemented control with a compact panicle; pV3-2, transgenic plant harbouring the 28.5-kb V3 genomic segment with a spread panicle; pOE-1, transgenic plant overexpressing *OsLG1* with a spread panicle. (**e**) Expression of *OsLG1* in transgenic plants shown in **d** by quantitative RT–PCR at the heading stage. Control CL2 (left), pV3-2 (center) and pOE-1 (right). Values are means and s.d. of three independent experiments. (**f**,**g**) Panicle of introgression line CIL49 and cultivar C418 at the heading stage. Scale bars, 5 cm. (**h**,**i**) Micrograph of the boxed regions in **f** and **g**, respectively. Scale bars, 0.5 mm. (**j**) Expression of *OsLG1* at the panicle pulvinus of introgression line CIL49 with a spread panicle, and reduced expression in the same regions in the compact panicle cultivar C418. Values are means and s.d. of three independent experiments.

Preliminary genetic analysis of an F_2_ population (285 progeny) derived from the cross between YIL31 and Teqing suggested that the spread panicle phenotype was controlled by a single dominant gene. Genetic linkage analysis with 1,368 F_2_ progeny revealed that the functional mutation (FM) of the target gene was located between markers M1 and M2 on chromosome 4 (Fig. 2a). Then, we used a much larger F_2_ population, consisting of 15,198 plants, and delimited the FM to a 3.3-kb region between the M5 and M6 markers (Fig. 2b).

M5 and M6 were used to identify a single BAC clone (YJ0510607) from the YJCWR genomic BAC library (Fig. 2c). Sequence analysis indicated that the fragment contained non-coding DNA as no predicated gene sequence was found within the 3.3-kb region. Two subcloned segments V1 (11.6-kb) and V2 (13.7-kb), harbouring the 3.3-kb region, were used to transform *japonica* cultivar Zhonghua 17 (with a compact panicle). All 44 independently generated transgenic lines (19 V1 lines and 25 V2 lines) still showed the compact panicle phenotype similar to Zhonghua 17, indicating that complementation had not occurred.

Further sequence analysis of the surrounding region revealed that the *OsLG1* gene, a previously reported SBP-domain transcription factor that controls rice ligule development^18^, is located 10 kb from the finely mapped region. To test whether *OsLG1* is responsible for the spread panicle phenotype, we introduced a 28.5-kb genomic segment (V3) harbouring the entire 3.3-kb fine-mapping region and the *OsLG1* into Zhonghua 17 (Fig. 2c). All nine independently generated transgenic lines showed full complementation with a spread panicle phenotype similar to that of YIL31 (Fig. 2d).

We sequenced the coding region of *OsLG1* and found no sequence variation between YIL31 and Teqing. We then compared the sequences of the 3.3-kb fine-mapping region between YIL31 and Teqing, and identified 12 SNPs and one 6-bp insertion/deletion variations (Supplementary Fig. S2). As the 3.3-kb region contained non-coding DNA, it is possible that these sequence variations in the 3.3-kb-mapping region may affect the *OsLG1* expression contributing to the transition of panicle architecture. Consistent with this speculation, we found that the mRNA expression level of *OsLG1* was much higher at the panicle pulvinus in the transgenic plants (spread panicle phenotype), compared with the control plant (compact panicle phenotype; Fig. 2e). Further confirmation was achieved by overexpressing the *OsLG1*-coding region, driven by the strong constitutive ubiquitin promoter, which induced the spread panicle phenotype in Zhonghua 17 transgenic lines (Fig. 2d,e). These results demonstrated that strong expression of *OsLG1* led to spread panicle architecture.

In addition to YIL31, we identified another introgression line CIL49 derived from a cross between YJCWR and compact panicle *japonica* cultivar C418. The CIL49 displayed similar spread panicle as YIL31 (Fig. 2f,g), and also had a panicle pulvinus that caused the larger angle between the panicle branch and main stem (Fig. 2h,i). Genotypic analysis also showed that the YJCWR segment was present in the long arm of chromosome 4 in CIL49. Sequence analysis indicated that no variation existed in *OsLG1*, whereas the 12 SNPs were present in the 3.3-kb region between CIL49 and C418. Quantitative reverse transcriptase (RT)-PCR analysis showed that *OsLG1* expression at the panicle pulvinus in CIL49 (spread panicle phenotype) was higher than that in C418 (compact panicle phenotype; Fig. 2j, Supplementary Table S2).

Taken together, these data suggest that variations in the 3.3-kb-mapping region of cultivated rice, located 10-kb upstream from the *OsLG1* translation start site, decreased the *OsLG1* gene expression at the panicle pulvinus, consequently leading to the transition from spread to compact panicle.

### An SNP caused a compact panicle in domesticated rice

**Figure 3:**
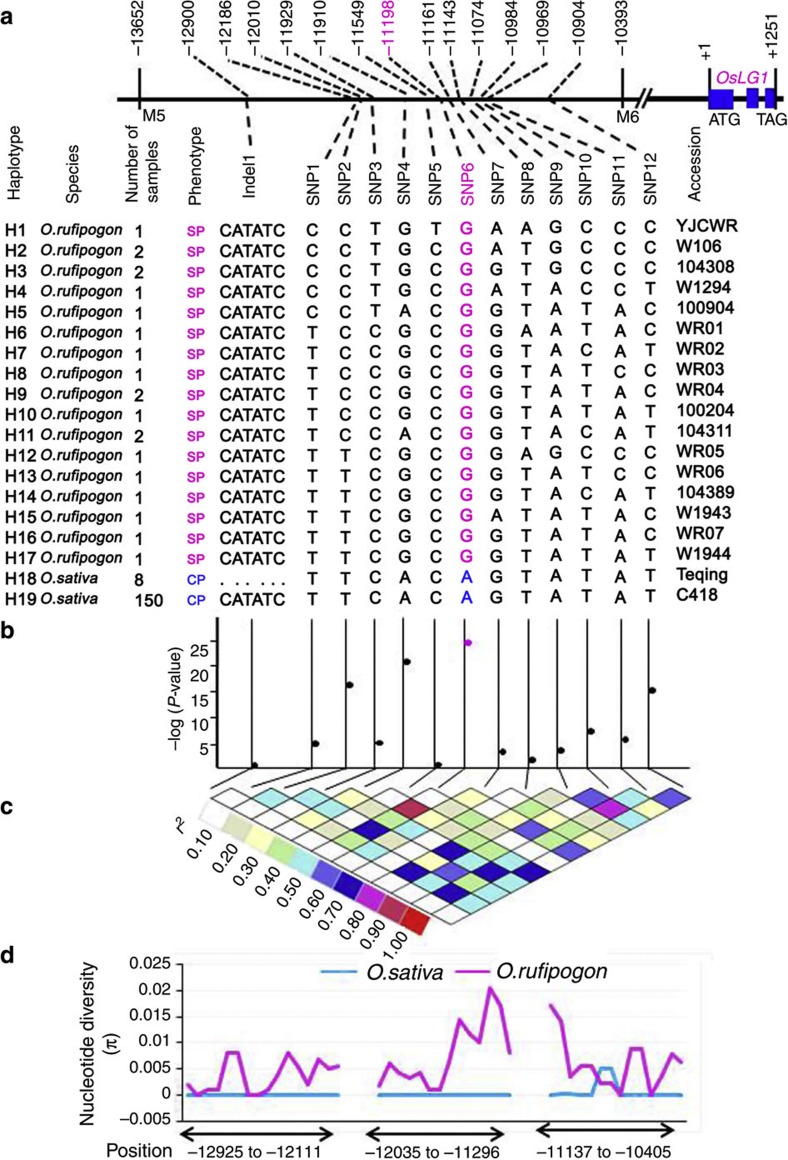
Haplotype analysis and association mapping in the 3.3-kb upstream regulatory region of *OsLG1*. (**a**) Haplotype analysis of the 3.3-kb upstream regulatory region with 21 accessions of wild rice and 158 domesticated cultivars. SP, spread panicle; CP, compact panicle. (**b**) Association testing of 13 variants in the 3.3-kb finely mapped region of *OsLG1*. Black dots represent 12 variations; pink dot represents the proposed functional variant site (SNP6). (**c**) Triangle matrix of pairwise linkage disequilibrium. (**d**) The comparison of nucleotide diversity (π) between wild and cultivated rice. The position is the physical position immediately upstream of the translation start site of the *OsLG1* gene.

To determine which mutation was associated with the phenotype, we sequenced the 3.3-kb fine-mapping region of 158 compact panicle cultivars from 17 countries and 21 spread panicle accessions of *O. rufipogon* (Supplementary Table S1). We found that the 21 accessions of wild rice clustered in 17 haplotypes (H1–H17), whereas the 158 cultivars clustered in only 2 haplotypes (H18 and H19; Fig. 3a). The haplotype H18 included Teqing and other seven cultivars, whereas the haplotype H19 included all the remaining 150 cultivars, suggesting that the *OsLG1* allele was strongly selected in cultivated rice. An association test with panicle architecture phenotypes and the 12 SNPs and one 6-bp insertion/deletion identified in wild and cultivated rice revealed that the strongest signal was present at the SNP6 site (*P*=1.2 × 10^−26^; Fig. 3b). Medium signals were detected at the SNP2, SNP4 and SNP12, which were in high linkage disequilibrium with the SNP6 (*r*^*2*^>0.6; Fig. 3c). Further sequences comparisons demonstrated that the nucleotide at the SNP6 site of all the 21 wild rice accessions is G, whereas that of all the 158 cultivars is A (Fig. 3a). This nucleotide change, G to A, at the SNP6 site was completely consistent with the transition of panicle architecture. These results suggested that the SNP6 mutation in the regulatory region was responsible for compact panicle architecture of cultivated rice.

To test whether the 3.3-kb regulatory region had been a target for artificial selection during rice domestication, we analysed the signature of selection in 3.3 kb sequences from a panel of 19 wild rice (*O. rufipogon*) and 62 cultivated varieties. The nucleotide diversity was nearly zero in the cultivated rice, which is significantly lower than that of wild rice (Fig. 3d). This result indicated that the 3.3-kb regulatory region had experienced strongly artificial selection during rice domestication.

### Phylogenetic analysis of *OsLG1*

**Figure 4:**
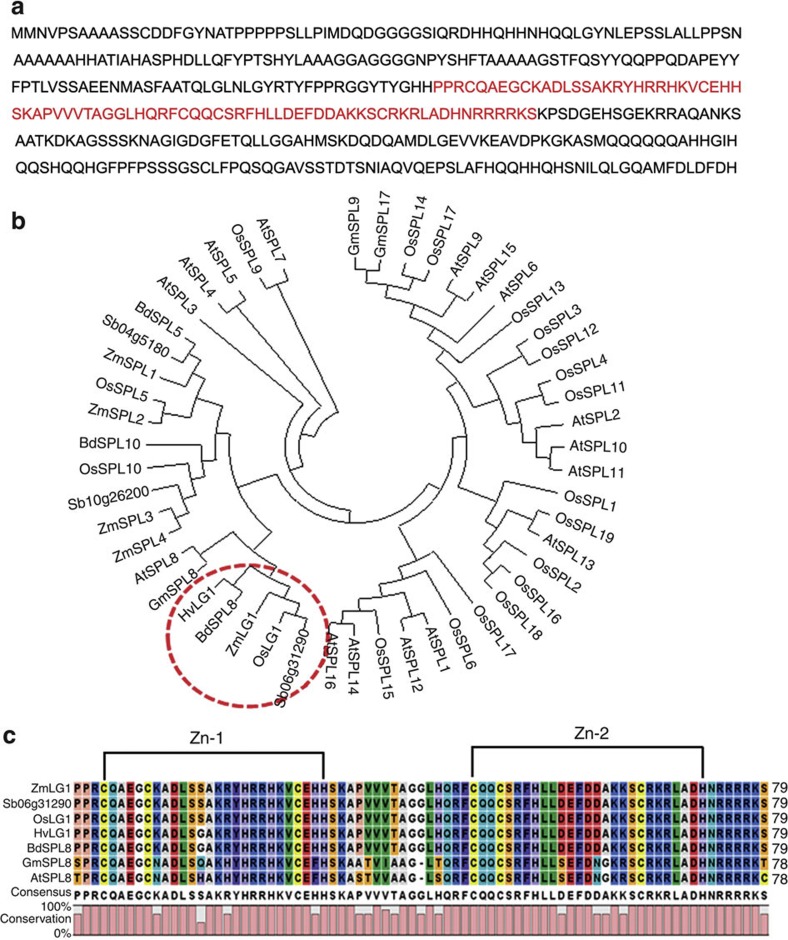
Phylogenetic analysis of OsLG1. (**a**) Predicted amino-acid sequence of OsLG1 with the SBP domain shown in red font. (**b**) MEGA5 neighbor-joining tree was inferred from the amino-acid sequences of the OsLG1 homologues among other plants. *At*, *Arabidopsis thaliana*; *Gm*, *Glycine max*; *Zm*, *Zea mays*, *Sb*, *Sorghum bicolor, Bd*, *Brachypodium distachyon*, *Hv*, *Hordeum vulgare.* (**c**) Homology analysis of SBP domains of putative orthologs for the *OsLG1* clade, showing full identity of *OsLG1* with genes from sorghum and maize. The SBP domain includes the Zn-1 and Zn-2 domains.

The *OsLG1* gene encodes the SBP-domain transcription factor *OsSPL8,* which encodes 416 amino acids (Fig. 4a). We retrieved through BLASTp searches using the full-length protein sequence of OsLG1 as a query against the non-redundant protein database ( http://www.ncbi.nlm.nih.gov/BLAST/), and identified 14 putative homologues in other crop plants. Phylogenetic analysis of these 14 putative homologues plus 35 SBP-domain genes previously identified in rice and *Arabidopsis* indicate that *OsLG1* is most closely related to genes found in other monocots, including sorghum (Sb06g31290), maize (*ZmLG1*)^20^, barley (*HvLG1*) and *Brachypodium* (*BdSPL8*; Fig. 4b). The SBP domains were highly conserved between OsLG1 and its homologues in other crop plants, noting that the predicted SBP domains of OsLG1, Sb06g31290 and ZmLG1 were completely identical (Fig. 4c).

### Expression pattern of *OsLG1*

**Figure 5:**
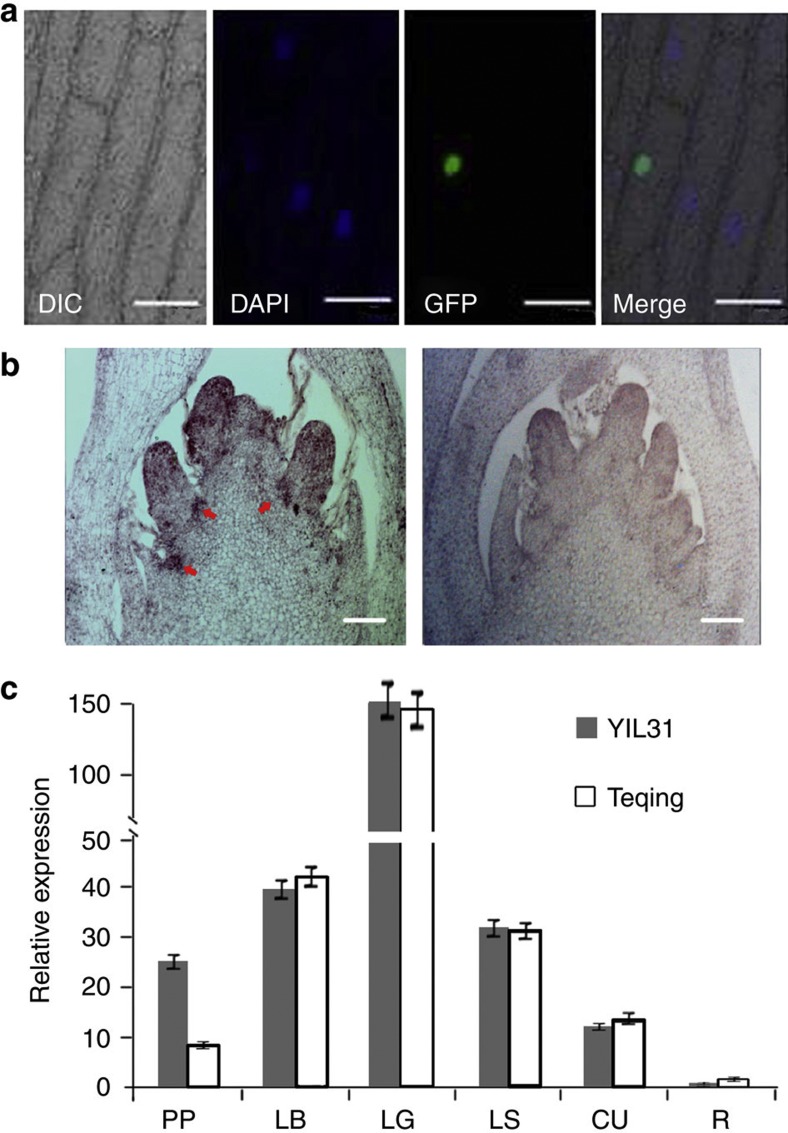
Subcellular localization and expression pattern of the *OsLG1* gene. (**a**) Subcellular localization of OsLG1. The *OsLG1–GFP* fusion gene under the control of the *CaMV35S* promoter was expressed transiently in onion epidermal cells. Scale bars, 100 μm. (**b**) *OsLG1* gene expression revealed by mRNA *in situ* hybridization in the young panicle of YIL31. Arrowheads indicate *OsLG1* expression in the panicle pulvinus. Left, antisense probe; right, sense probe (control). Scale bars, 100 μm. (**c**) Expression of *OsLG1* in different tissues of YIL31 and Teqing by quantitative RT–PCR at the late panicle development stage. PP, panicle pulvinus; LB, leaf blade; LG, leaf ligule; LS, leaf sheath; CU, culm; R, root. Values are means and s.d. of three independent experiments.

The *OsLG1* gene belongs to the SPL transcription factor family related to the plant architecture and inflorescence development^21^,^22^,^23^,^24^,^25^. When we introduced the *OsLG1–GFP* (green fluorescent protein) fusion gene under control of the *CaMV35S* promoter into the onion epidermal cells, we found that the OsLG1–GFP fusion protein localized to the nucleus (Fig. 5a), as predicted for a functional transcriptional factor. RNA *in situ* hybridization results showed that *OsLG1* was strongly expressed at the panicle pulvinus (Fig. 5b), which is consistent with its role of controlling cellular development in that region.

Using quantitative RT–PCR, we found that *OsLG1* mainly expressed at the panicle pulvinus, leaf sheath, ligule, leaf blade and culm (Fig. 5c). *OsLG1* expression at the panicle pulvinus was higher in YIL31 and CIL49 than that in Teqing and C418 (Fig. 5c). To further investigate whether the *OsLG1* gene expression changes result from the methylation level differences of the *OsLG1* promoter region, we collected the panicle pulvinus of YIL31 and Teqing and performed bisulfite sequencing to compare DNA methylation levels of the 1.4-kb promoter region upstream the translation start site. Significant differences of methylation were observed at Cyt_241 and Cyt_244 cytosine site, with higher levels of methylation in Teqing (~83% at the Cyt_241 site, ~40% at the Cyt_244 site) compared with YIL31 (~40% at the Cyt_241 site, ~16% at the Cyt_244 site; Supplementary Fig. S3, Supplementary Table S3), implying that the DNA methylation levels in the promoter region of *OsLG1* might affect the *OsLG1* gene expression change between Teqing and YIL31 in the panicle pulvinus.

## Discussion

In this paper, we demonstrated that the *OsLG1* is a key gene controlling rice panicle architecture. Association analysis indicates that selection of an SNP residing in the *cis*-regulatory region of the *OsLG1* gene was responsible for the transition from a spread panicle typical of ancestral wild rice to the compact panicle of present cultivars during rice domestication.

A rice leaf consists of a leaf blade, a leaf sheath and a laminar joint that contains a pair of auricles and the ligules. Previous study showed that *OsLG1* regulated the rice ligule development by characterization of a T-DNA knockout mutant^18^. The observations of dual roles for *OsLG1* in regulating ligule development and panicle architecture indicate that *OsLG1* has pleiotropic effects. The *ZmLG1* gene, presumably orthologous to *OsLG1* (Fig. 4), also controls the branch angle of the tassel and is required for the ligule development in maize^20^,^26^. Knockout mutants of *ZmLG1* result in a more compact tassel than their wild-type counterparts and loss of the ligules in maize, indicating that the pleiotropic gene functions of both *OsLG1* and *ZmLG1* are conserved between these species.

The nucleotide diversity of cultivated rice in the 3.3-kb regulatory region of the *OsLG1* gene was significantly lower compared with the ancestral species, *O. rufipogon* (Fig. 3d). Very recently, a separate work also indicated that the upstream regulatory sequences of the *OsLG1* gene were responsible for the compact panicle of domesticated rice, although the FM was not identified^27^. Furthermore, the whole-genome sequencing study showed that *OsLG1* was under strong artificial selection^28^. These results suggested that the *OsLG1* region experienced artificial selection during the rice domestication.

Although several genes including *qSH1* and *TB1*, controlling domestication-related traits with the causal mutations in the *cis*-regulatory region, had been identified^6^,^29^,^30^,^31^, the advantage of the selection of *cis*-regulatory mutation during crop domestication is poorly known. *OsLG1* was shown to have pleiotropic effects with regard to spread panicle architecture and normal ligule development in rice^25^,^27^ (Fig. 2). The ligule is an important functional organ as it can prevent rainwater and dust from entering the leaf sheath. Although a spread panicle is beneficial for wild rice, the compact panicle is a desirable trait for cultivation purposes. The domestication of rice, therefore, required a transition in panicle architecture, from spread to compact, without changing the ligule. Either intentionally or fortuitously, mutations in the *OsLG1* regulatory region, reducing its expression specifically at the panicle pulvinus, were selected by ancient humans for a favourable compact panicle architecture in domesticated rice while maintaining normal ligule formation.

Crop domestication is the genetic modification of a wild species to generate a new form of a crop altered to meet human needs^32^. During domestication and improvement of crops, problematic agronomic traits associations often occur, which result from pleiotropic effects of the target gene on other traits, or tight linkage of genes that control independent traits (known as ‘linkage drag’), which pose penalties on crop production^33^. Strategies targeting changes to the favourable gene function circumventing undesirable agronomic trait associations has been a difficult problem for crop breeders. Our findings indicate that sequence variance in the *cis*-regulatory region finely tune spatial expression levels of pleiotropic gene effects, possibly by changing the methylation status of the promoter region of the target gene, and consequently break the negative agronomic trait association resulting from pleiotropic gene effects. This novel discovery is very important for understanding the mechanism of crop domestication and will improve the molecular breeding of crops in the future.

## Methods

### Plant materials

Teqing, an elite *indica* cultivar (*O. sativa* L.), and YJCWR, a common wild-rice accession (*O. rufipogon* Griff.) collected as rhizomes from Yujiang province, China, were used as recurrent and donor parents in a backcrossing programme, respectively. The F_1_ plant derived from the cross between Teqing and YJCWR were backcrossed three times consecutively with Teqing to generated introgression population. We selected an introgression line YIL31 displaying a spread panicle phenotype to backcross with Teqing. The F_2_-segregating population was used for genetic analysis and fine mapping. A set of 158 diverse *O. sativa* and 21 accessions of wild rice from 17 countries used in this study are listed in Supplementary Table S1.

### Primers

The primers used in this study are listed in Supplementary Table S2.

### Complementation test

The BAC clone YJ0510607, containing the *OsLG1* gene, was identified from the BAC library of YJCWR^34^. The 11.6-kb and 13.7-kb fragments, harbouring the 3.3-kb-mapping region, were inserted into the binary vector pCAMBIA1300 to form pV1 and pV2, respectively. These two plasmid constructs were introduced to *Agrobacterium tumefaciens* strain LBA4404 and subsequently transferred into the *japonica* cultivar Zhonghua 17, which had compact panicle. The construct pV3 contained a 28.5-kb segment harbouring the whole fine-mapping region and the *OsLG1* gene. The pV3 were mixed with pCAMBIA1300 and transferred into Zhonghua 17 using a helium biolistic device (Bio-Rad PDS-1000). To generate the overexpression construct pOE, the ORF of *OsLG1* was amplified from first-strand cDNA of YIL31. The construct drove the expression of *OsLG1* cDNA under the control of the ubiquitin promoter.

### Subcellular localization

Subcellular localization of OsLG1 was determined using the coding sequence of a GFP fused in-frame to the *OsLG1*-coding sequence and transcribed from a *CaMV35S* promoter. The resulting plasmid was bombarded into onion epidermal cells using a helium biolistic device (Bio-Rad PDS-1000). We examined the bombarded tissues with a confocal laser-scanning microscope (Carl Zeiss LAM510).

### RNA *in situ* hybridization experiment

The rice young panicles of YIL31 and Teqing were collected and fixed with 4% (w/v) paraformaldehyde at 4 °C overnight, followed by a series of dehydration and infiltration, and were embedded in paraffin (Paraplast Plus, Sigma). The tissues were sliced into 8-μm sections with a microtome (Leica RM2145). The 396-bp 3′-region of *OsLG1* cDNA was subcloned into the pSK vector and used as the template to generate sense and antisense RNA probes. Digoxigenin-labelled RNA probes were prepared using a DIG Northern Starter Kit (Catalogue number 2039672, Roche) according to the manufacturer’s instruction. Slides were observed under bright field through a microscope (Leica DMR) and photographed with a micro colour charge-coupled device camera (Apogee Instruments).

### Quantitative RT–PCR

We extracted total RNAs using an RNeasy Plant Mini Kit (Qiagen). First-stand cDNA synthesis was done with BcaBEST RNA PCR Kit (TaKaRa). Real-time RT–PCR was done on the ABI Prism 7900 Sequence Detection System (Applied Biosystems). Diluted cDNA was amplified using the SYBRGreen Master Mix (Applied Biosystems). We normalized the levels of *OsLG1* transcripts by endogenous 18S rRNA transcripts amplified with primers 18SF and 18SR. Each set of experiments was repeated three times, and the relative quantification method (2^−ΔΔC_T_^ (DDCT)) was used to evaluate quantitative variation.

### Bisulfite genomic sequencing

We collected the base of panicle branch of YIL31 (containing the panicle pulvinus) and Teqing (without the panicle pulvinus), and then isolated the genomic DNA. The DNA was bisulfite treated using the EZ DNA Methylation-Gold kit. The 1.4-kb promoter region upstream the translation start site of *OsLG1* gene was amplified using bisulfite primers and cloned into the pCR-4 vector. Each fragment was sequenced in at least 30 clones.

### Association mapping

Association testing was performed with Fisher’s exact test in R, as Fisher’s exact test can be powerful for the binary trait such as rice panicle branch angle^35^.

## Author contributions

C.S. designed and supervised this study. Z.Z. performed most of the experiments. L.T., Y.F., F.L., F.W., J.W. and T.M. performed some of the experiments. C.S., Z.Z., H.C. and D.X. analysed the data and wrote the paper.

## Additional information

**Accession codes**: Sequence data have been deposited in GenBank/EMBL/DDBJ under Accession numbers JX462783, JX462784.

**How to cite this article:** Zhu, Z. *et al.* Genetic control of inflorescence architecture during rice domestication. *Nat. Commun.* 4:2200 doi: 10.1038/ncomms3200 (2013).

## Supplementary Material

Supplementary Information Supplementary Figures S1-S3 and Supplementary Tables S1-S3
