# Supplementary material for: Genetic control of inflorescence architecture during rice domestication
Source: Nat Commun. 2013 Jul 25;4:2200. doi: 10.1038/ncomms3200 (PMC3731664; doi:10.1038/ncomms3200)
Supplement: Supplementary Information — Supplementary Figures S1-S3 and Supplementary Tables S1-S3 [file ncomms3200-s1.pdf]

Supplementary Information:

Genetic control of inflorescence architecture during rice domestication

Zuofeng Zhu[1], Lubin Tan[1], Yongcai Fu[1], Fengxia Liu[1], Hongwei Cai[1], Daoxin Xie[2], Feng Wu[3], Jianzhong Wu[4], Takashi Matsumoto[4], Chuanqing Sun[1]*

[1]State Key Laboratory of Plant Physiology and Biochemistry, National Center for Evaluation of Agricultural Wild Plants (Rice), China Agricultural University, Beijing 100193, China.

[2]School of Life Sciences, Tsinghua University, Beijing 100084, China.

[3]Institute of Botany, Chinese Academy of Sciences, Beijing 100093, China.

[4]National Institute of Agrobiological Sciences, Tsukuba 305-8602, Japan.

[*]Correspondence should be addressed to C.S. (suncq@cau.edu.cn)

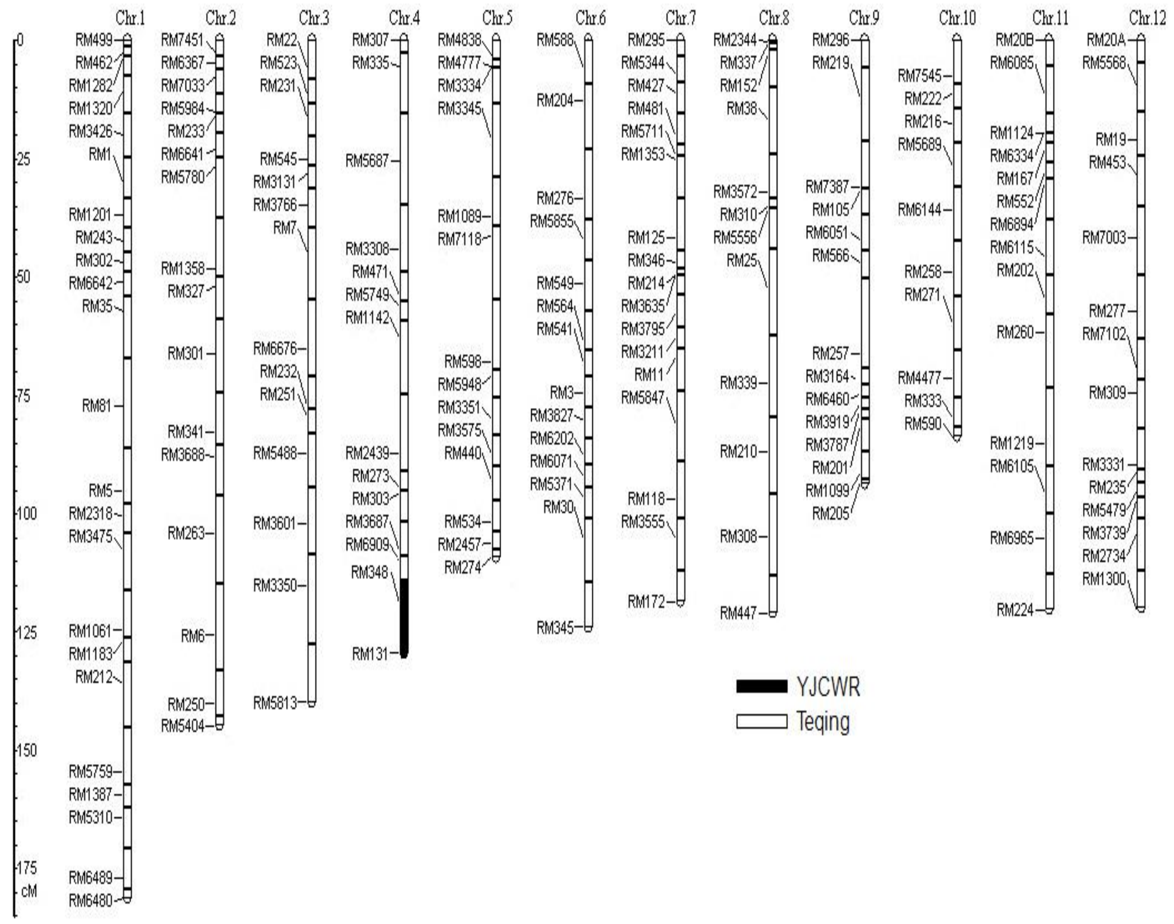

Supplementary Figure S1. Graphical genotypes of YIL31. The black regions indicate the homozygous regions for Yuanjiang common wild rice (*O. rufipogon*). The white regions indicate the homozygous regions for Teqing (*O. sativa* ssp. *indica*).

```
YJCWR     1    GTTCATTTAAATAATTATCACACATTATTCACCAGCCATTCAACATAAAAGAACAGGGAA 60
Teqing    1    GTTCATTTAAATAATTATCACACATTATTCACCAGCCATTCAACATAAAAGAACAGGGAA 60

YJCWR     61   GAAGAAGCAACTATATGGATTGTGTCCCCCTGATGATAATAGATACTATGTACTCTCTAG 120
Teqing    61   GAAGAAGCAACTATATGGATTGTGTCCCCCTGATGATAATAGATACTATGTACTCTCTAG 120

YJCWR     121  CCCGATATTTAATTATAAAGGGGGTGATCTATAGGTGCAAATGAAGATGGTGACCTGGTA 180
Teqing    121  CCCGATATTTAATTATAAAGGGGGTGATCTATAGGTGCAAATGAAGATGGTGACCTGGTA 180

YJCWR     181  TGATCAATGTGGACAAGTGGGACATAATCATAAAAAGGAGTCCAGATACTGACCAATGCA 240
Teqing    181  TGATCAATGTGGACAAGTGGGACATAATCATAAAAAGGAGTCCAGATACTGACCAATGCA 240

YJCWR     241  AATGCTGCGTCAGAGATTGTGATAGATGGAGGGCCAGATCGAGTCATGACCGGGCTGTGC 300
Teqing    241  AATGCTGCGTCAGAGATTGTGATAGATGGAGGGCCAGATCGAGTCATGACCGGGCTGTGC 300

YJCWR     301  ATGCTACTAGCTATTACTCAAGGAGTACTGATCCAGTAGTACTACATGGGAAGGAATTAG 360
Teqing    301  ATGCTACTAGCTATTACTCAAGGAGTACTGATCCAGTAGTACTACATGGGAAGGAATTAG 360

YJCWR     361  GAATAACAGGCAGCATAAGGCCTCAATGACATGAAAATCTTGTATGATGTATTAATTTGG 420
Teqing    361  GAATAACAGGCAGCATAAGGCCTCAATGACATGAAAATCTTGTATGATGTATTAATTTGG 420

YJCWR     421  GTGTAAGATCACTGATCACAATGACCTGTTAAAGTTAAACACCTATTTCTCTGAAAGTTT 480
Teqing    421  GTGTAAGATCACTGATCACAATGACCTGTTAAAGTTAAACACCTATTTCTCTGAAAGTTT 480

YJCWR     481  CTCGAAAACGTGTGGGGTTGCTGCATATAAATCCACCACTGTCTCGTCCTGCTCTGCTCA 540
Teqing    481  CTCGAAAACGTGTGGGGTTGCTGCATATAAATCCACCACTGTCTCGTCCTGCTCTGCTCA 540

YJCWR     541  CTTTCAGGACGTGTCCTTGGTGATCAAAATTTCAACAATATATCTACATATTGCCCATAT 600
Teqing    541  CTTTCAGGACGTGTCCTTGGTGATCAAAATTTCAACAATATATCTACATATTGCCCATAT 600

YJCWR     601  CACCATCTGCATGATTGAAACCTTGGTAAATTTTAATTTTACTTGTGTTCAGTTGATCAG 660
Teqing    601  CACCATCTGCATGATTGAAACCTTGGTAAATTTTAATTTTACTTGTGTTCAGTTGATCAG 660

YJCWR     661  TTCACATGTGTCTGTACCAGTGAAATGGAGTAAAAGAGTGACAGTACCCGGCCGATCGAT 720
Teqing    661  TTCACATGTGTCTGTACCAGTGAAATGGAGTAAAAGAGTGACAGTACCCGGCCGATCGAT 720
                                                 InDel1
YJCWR     721  ATGATATGATCGAGCCGCTCGCGGTCCCCTACATATCGATCTATCTATCTATCTATCGCA 780
Teqing    721  ATGATATGATCGAGCCGCTCGCGGTCCCCTA------GATCTATCTATCTATCTATCGCA 774

YJCWR     781  CAAAAGCTGCCGAAAATTGAAACAATTATTGGGATGATAGGACCAGCAATGTAAGCTGCA 840
Teqing    775  CAAAAGCTGCCGAAAATTGAAACAATTATTGGGATGATAGGACCAGCAATGTAAGCTGCA 834

YJCWR     841  TCAGAGGCGAGATTGATCGGTTGATCAGAGGGCGATATGTTTGCGCCGATTGATTCGAGA 900
```

Teqing 835 TCAGAGGCGAGATTGATCGGTTGATCAGAGGGCGATATGTTTGCGCCGATTGATTCGAGA 894

YJCWR 901 TTTTGTGGACTTCAAGGGGGTTTTCCTGTGCTGTGCTGCCTTTTCGCTCATTCGAATCAG 959
Teqing 895 TTTTGTGGACTTCAAGGGGGTTTTCCTGTGCTGTGCTGCCTTTTCGCTCATTCGAATCAG 954

YJCWR 960 AGCAGCTGCAACGCATGCATGCGCTTCTGAAACCTGCAAACGCCTGACCCTCAATGCTCA 1019
Teqing 955 AGCAGCTGCAACGCATGCATGCGCTTCTGAAACCTGCAAACGCCTGACCCTCAATGCTCA 1014

YJCWR 1020 ATGCAACTTTGAGTATATTATCGTGCACGTGGACGGCACAGCGATCCCAATCAGGTTTTG 1079
Teqing 1015 ATGCAACTTTGAGTATATTATCGTGCACGTGGACGGCACAGCGATCCCAATCAGGTTTTG 1074

YJCWR 1080 GTGGATAGAGTTTGTCTCATTCGATCCAATTTATGGCTCTGTAAATGCTGTTTTTGCAGA 1139
Teqing 1075 GTGGATAGAGTTTGTCTCATTCGATCCAATTTATGGCTCTGTAAATGCTGTTTTTGCAGA 1134

YJCWR 1140 GAAAAATTCAGTTCATTGTTTTCCCTGAATCACTTAATTAATTAATCTCGTAGAGGTATA 1199
Teqing 1135 GAAAAATTCAGTTCATTGTTTTCCCTGAATCACTTAATTAATTAATCTCGTAGAGGTATA 1194

YJCWR 1200 TTGGAAAAAAAACAAAGAAAAAAAAGGTAGGAGATCGATCAAGAAGGATGGGCGGCGAAA 1259
Teqing 1195 TTGGAAAAAAAACAAAGAAAAAAAAGGTAGGAGATCGATCAAGAAGGATGGGCGGCGAAA 1254

YJCWR 1260 GGTCAGATTTATTCGGATTTAATTCGTTTTGAAAATTGTGCAAGACCGGCTTGAAAAACA 1319
Teqing 1255 GGTCAGATTTATTCGGATTTAATTCGTTTTGAAAATTGTGCAAGACCGGCTTGAAAAACA 1314

YJCWR 1320 ACTCTCAGCTACAAGGAAAATCTTCACGTCCTTTAACACTTATTGAAATCAATATGTTTT 1379
Teqing 1315 ACTCTCAGCTACAAGGAAAATCTTCACGTCCTTTAACACTTATTGAAATCAATATGTTTT 1374

YJCWR 1380 GACAAGTTATGTAAGTGAGAAATCGATTAATCAATGCGATGTTGTACATCATGTGGAAGA 1439
Teqing 1375 GACAAGTTATGTAAGTGAGAAATCGATTAATCAATGCGATGTTGTACATCATGTGGAAGA 1434

SNP1

YJCWR 1440 AATTGCCCTTTGTTTTCTTGTTTAATCGGTGCACTACATTACTACTGGTAATGAATTCTT 1499
Teqing 1435 AATTGCCCTTTGTTTTCTTGTTTAATTGGTGCACTACATTACTACTGGTAATGAATTCTT 1494

YJCWR 1500 GGCTCTCTAGTTCATTAATTATAGGAAATTACTACATTGTCGTGGCAGGCTAAGGATTGT 1559
Teqing 1495 GGCTCTCTAGTTCATTAATTATAGGAAATTACTACATTGTCGTGGCAGGCTAAGGATTGT 1554

YJCWR 1560 TCTTTAACCTTTTTACCGTGGACGAAAAGAAACACAGAGAATTGAAGGAAAGGAAAAAAA 1619
Teqing 1555 TCTTTAACCTTTTTACCGTGGACGAAAAGAAACACAGAGAATTGAAGGAAAGGAAAAAAA 1614

YJCWR 1620 AAAAGGAGGCAACAACGCCACAAGCAGGAAGATCGAATGTACAGCAGGCTACCAAATAGT 1678
Teqing 1615 AAAAGGAGGCAACAACGCCACAAGCAGGAAGATCGAATGTACAGCAGGCTACCAAATAGT 1674

SNP2 **SNP3**

YJCWR 1679 CGATTAAGAAAAGGCACTATTTAATGTGACTATTCCCCTATTTGCCTTGGTATTCATGCC 1738
Teqing 1675 TGATTAAGAAAAGGCACTATTTAATGTGACTATTCCCCTATTTGCCTTGGTACTCATGCC 1734

```
YJCWR    1739 AAAAATAGAAGAATTAACAAGTGATGGTTATTTAACTGACCGGTGGGCCCGGCGCTCTTG 1798
Teqing   1735 AAAAATAGAAGAATTAACAAGTGATGGTTATTTAACTGACCGGTGGGCCCGGCGCTCTTG 1794

                                                            SNP4
YJCWR    1799 TCTGCCCTGGTCAGTTACTATTACCAGCTGCTTTTTACCCTGGGCAACTCTGTTCACCCC 1858
Teqing   1795 TCTGCCCTGGTCAGTTACTATTACCAGCTGCTTTTTACCCTGAGCAACTCTGTTCACCCC 1854

YJCWR    1859 AAAGTATTGCATCCGAGCCCAAGCCATGGACAAGCACACGTGGTCTTTCATGCCACTGCC 1918
Teqing   1855 AAAGTATTGCATCCGAGCCCAAGCCATGGACAAGCACACGTGGTCTTTCATGCCACTGCC 1914

YJCWR    1919 ACTTTGTACGGCAGTTTGCGGCTACATCGGATCGTCTGCGTCGCCTATGTTATGCTGCCA 1978
Teqing   1915 ACTTTGTACGGCAGTTTGCGGCTACATCGGATCGTCTGCGTCGCCTATGTTATGCTGCCA 1974

YJCWR    1979 TATTCATAGTAAGAGTAGTACTAGTGCGGAATTCGAATTAGACCGCGCATGGAGAAAAGA 2038
Teqing   1975 TATTCATAGTAAGAGTAGTACTAGTGCGGAATTCGAATTAGACCGCGCATGGAGAAAAGA 2034

YJCWR    2039 TCCAAAGCGTCTGTTTGGGGGAGCTTCTAGTTGCTGTAGCTACTCCTAAAATCAGAAGCT 2098
Teqing   2035 TCCAAAGCGTCTGTTTGGGGGAGCTTCTAGTTGCTGTAGCTACTCCTAAAATCAGAAGCT 2094

                 SNP5
YJCWR    2099 CCCTAAAACAGTCTAGCTTTTGGTCCAGATTTGAGAAGCTGTAATTGTAGAATCTAGAAA 2158
Teqing   2095 CCCCAAAACAGTCTAGCTTTTGGTCCAGATTTGAGAAGCTGTAATTGTAGAATCTAGAAA 2154

YJCWR    2159 ATGAACTAGCAGCCAGAAGCTGGGAAACCCAGCTTTTCCAGATTCTCAGAAGCTGGCTAC 2218
Teqing   2155 ATGAACTAGCAGCCAGAAGCTGGGAAACCCAGCTTTTCCAGATTCTCAGAAGCTGGCTAC 2214

YJCWR    2219 CAATCAACTGCTTCTTCGAATTTTAAGCTCCCTCAAACAGGCCCAAAGTCTTGTACATAG 2278
Teqing   2215 CAATCAACTGCTTCTTCGAATTTTAAGCTCCCTCAAACAGGCCCAAAGTCTTGTACATAG 2274

YJCWR    2279 ACAAGGAATATGAAATCTTTTGTTTGAAAACTTAAGTTAGGTATTAGCACGTGACATGAG 2338
Teqing   2275 ACAAGGAATATGAAATCTTTTGTTTGAAAACTTAAGTTAGGTATTAGCACGTGACATGAG 2334

                                         SNP6
YJCWR    2339 CCTACACGTCTATATCAGTTTAGCGAAGCGTATAGTGTTAAGATTAATTCAAAATGACAA 2398
Teqing   2335 CCTACACGTCTATATCAGTTTAGCAAAGCGTATAGTGTTAAGATTAATTCAAAATGACAA 2394

               SNP7
YJCWR    2399 CAACTCGTAAGGTTGAGAATGGGTTCTGACAATGGTCGGCTATGTACCTACAAAAGATCA 2458
Teqing   2395 CGACTCGTAAGGTTGAGAATGGGTTCTGACAATGGTCGGCTATGTACCTACAAAAGATCA 2454

                                                                  SNP8
YJCWR    2459 CAAAACATATCTCGATCAATTCATTGTGATATTTAATGCCCCCAAAGCAAATATATAAGT 2518
Teqing   2455 CAAAACATATCTCGATCAATTCATTGTGATATTTAATGCCCCCAAAGCATATATATAAGT 2514

                                                               SNP9     SNP10
YJCWR    2519 TTTCACATTTCCGCCGAATTCATCCAGAAACACATAATCGTGGAAAGACTAAGCCTATCG 2578
Teqing   2515 TTTCACATTTCCGCCGAATTCATCCAGAAACACATAATCGTGGAAAAACTAAGCCTATTG 2574
```

```
                         SNP11
YJCWR        2579 GGTACCGCCGCCACCGCGCCGGCCACGCGAGACCGGAAGCGCAGAAGATCCTGGCTATCC 2638
Teqing       2575 GGTACCGCCGCCAACGCGCCGGCCACGCGAGACCGGAAGCGCAGAAGATCCTGGCTATCC 2634

YJCWR        2639 ATGGACCACACAAGGCAGGGGCAGGCTCTTCCACAATCCCGGGTCTGGTGACATGAGCCA 2698
Teqing       2635 ATGGACCACACAAGGCAGGGGCAGGCTCTTCCACAATCCCGGGTCTGGTGACATGAGCCA 2694
                                                                   SNP12
YJCWR        2699 CAGTTGTGGCCCGAACAAAGTGACGTGTGTCGAGTCGAACGCCAGGCCCCGCGCACACAT 2758
Teqing       2695 CAGTTGTGGCCCGAACAAAGTGACGTGTGTCGAGTCGAACGCCAGGCCTCGCGCACACAT 2754

YJCWR        2759 GCATTGCACTGGCACCCGCTGCGCCTGCGCTGCATGCACTGCTGCGTCCCAGCATTTTCT 2818
Teqing       2755 GCATTGCACTGGCACCCGCTGCGCCTGCGCTGCATGCACTGCTGCGTCCCAGCATTTTCT 2814

YJCWR        2819 CCCCCTTCTTTTGTACCGGCCTCCCACACGCACCGCGCGTCCTCCTGCTGGCCAAAAAGC 2878
Teqing       2815 CCCCCTTCTTTTGTACCGGCCTCCCACACGCACCGCGCGTCCTCCTGCTGGCCAAAAAGC 2874

YJCWR        2879 CAAATGTTGTGCTCCAAACGGCCGGCGGTTCCGGCGGCTCGACCTCGACGCGACGTTGCT 2938
Teqing       2875 CAAATGTTGTGCTCCAAACGGCCGGCGGTTCCGGCGGCTCGACCTCGACGCGACGTTGCT 2934

YJCWR        2939 TTGCTTCGTGCTCGTGCATATACGGGTCGTTCTTTGCTTTTGCATATTTATATTGCAAAC 2998
Teqing       2935 TTGCTTCGTGCTCGTGCATATACGGGTCGTTCTTTGCTTTTGCATATTTATATTGCAAAC 2994

YJCWR        2999 GGTGCGTGTGTGATGTGTCGATCGAATCCTGGTCAAATATGGCGTACTTTATTCTCGCAG 3058
Teqing       2995 GGTGCGTGTGTGATGTGTCGATCGAATCCTGGTCAAATATGGCGTACTTTATTCTCGCAG 3054

YJCWR        3059 TTTGAGATCGATATGCTATTGCACCGAACGCTGCTCAGAGAGGGGGCTTTCTGACCAGCT 3118
Teqing       3055 TTTGAGATCGATATGCTATTGCACCGAACGCTGCTCAGAGAGGGGGCTTTCTGACCAGCT 3114

YJCWR        3119 GGTAGGCAGGGGTGCAAAAAACCAGAGTCTAATTTTATATGTACTCTAATCTAAATTCAT 3178
Teqing       3115 GGTAGGCAGGGGTGCAAAAAACCAGAGTCTAATTTTATATGTACTCTAATCTAAATTCAT 3174

YJCWR        3179 TAGCCTGATGATAGTATCCAGTAGCCACATAGCCAACAATTAGACAATTTTCTTTTTTAA 3238
Teqing       3175 TAGCCTGATGATAGTATCCAGTAGCCACATAGCCAACAATTAGACAATTTTCTTTTTTAA 3234

YJCWR        3239 TGAAAAATCAGGTTATTTTGG 3259
Teqing       3235 TGAAAAATCAGGTTATTTTGG 3255
```

Supplementary Figure S2. The variations in the 3.3 kb mapping region between Teqing and YIL31.

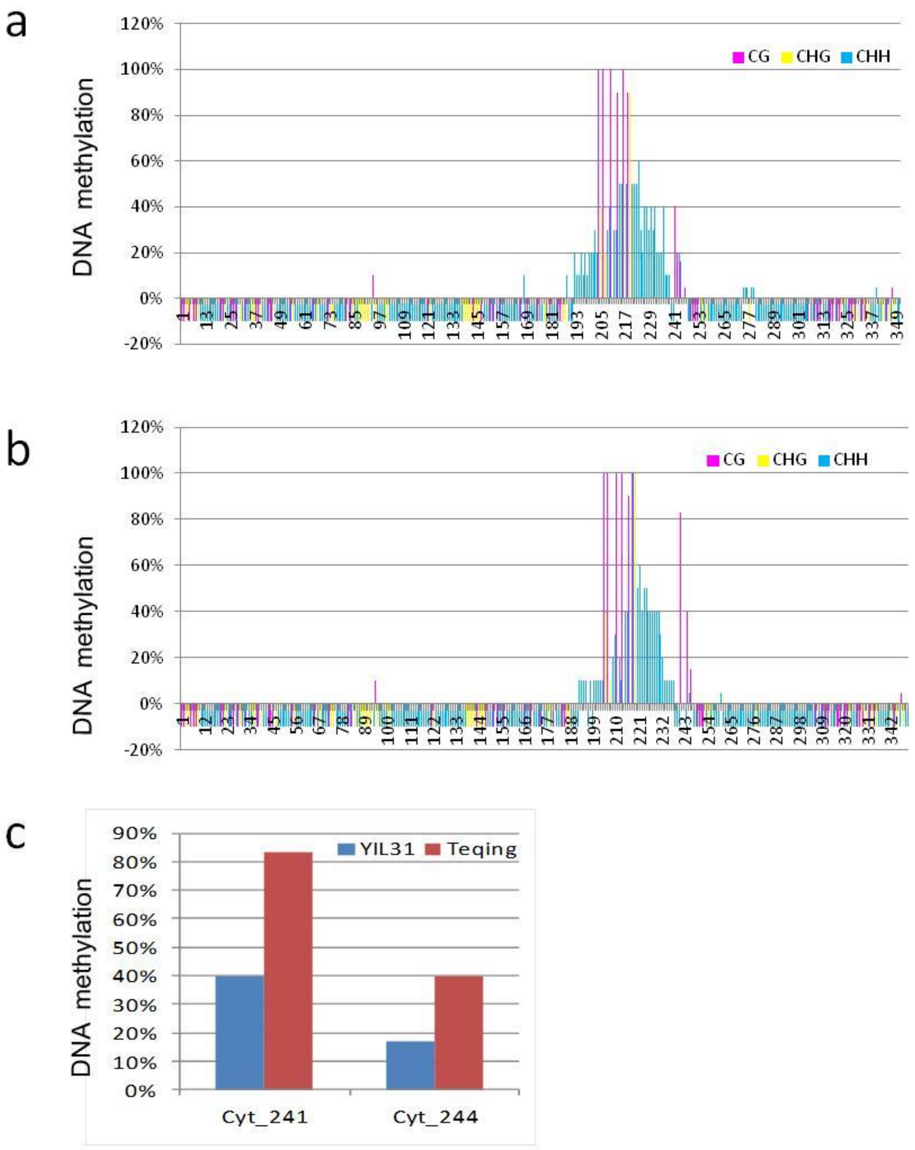

Supplementary Figure S3. Comparison of the methylation level of the promoter region of *OsLG1* gene between YIL31 and Teqing.

(a) and (b)The methylation level of the 1.4-kb promoter region of *OsLG1* gene in the branch pulvinus of panicle in YIL31 (a) and Teqing (b). The numbers under the graph indicate the position of the cytosine, which sequence position were listed in the **supplementary table 3**. (c) Comparison of methylation level at Cyt_241 and Cyt_244 cytosine site between YlL31 and Teqing.

Supplementary Table S1. Materials used in this study

Name	Origin	Species name	Phenotype	Haplotype
YJCWR*	Yunnan,China	*O. rufipogon*	spread	H1
W106*	India	*O. rufipogon*	spread	H2
IRGC105493	Myanmar	*O. rufipogon*	spread	H2
IRGC104308*	Myanmar	*O. rufipogon*	spread	H3
IRGC100926	Myanmar	*O. rufipogon*	spread	H3
W1294*	Philippines	*O. rufipogon*	spread	H4
IRGC100904*	Thailand	*O. rufipogon*	spread	H5
YD4-50*	Jiangxi, China	*O. rufipogon*	spread	H6
YD4-151*	Jiangxi, China	*O. rufipogon*	spread	H7
WRCAU1*	Guangdong, China	*O. rufipogon*	spread	H8
YD2-0261*	Guangxi, China	*O. rufipogon*	spread	H9
YD2-0344	Guangxi, China	*O. rufipogon*	spread	H9
IRGC100204*	India	*O. rufipogon*	spread	H10
IRGC104311*	Thailand	*O. rufipogon*	spread	H11
IRGC104599	Sri Lanka	*O. rufipogon*	spread	H11
S9022*	Guangdong, China	*O. rufipogon*	spread	H12
S8021*	Guangdong, China	*O. rufipogon*	spread	H13
IRGC104389*	Thailand	*O. rufipogon*	spread	H14
W1943*	Jiangxi, China	*O. rufipogon*	spread	H15
WRCAU2*	Hunan, China	*O. rufipogon*	spread	H16
W1944*	Hunan, China	*O. rufipogon*	spread	H17
Teqing	China	*indica*	compact	H18
Sanbaili	China	*indica*	compact	H18
Esiniu	China	*indica*	compact	H18
Huangsiguizhan	China	*indica*	compact	H18
Shuusoushu*	China	*indica*	compact	H18
Keiboba*	China	*indica*	compact	H18
Deejiaohualuo*	China	*indica*	compact	H18
Hong cheuhzai*	China	*indica*	compact	H18
C418	China	*indica*	compact	H19
Baimaodao	China	*indica*	compact	H19
Xiangaizao 10	China	*indica*	compact	H19
Nantehao	China	*indica*	compact	H19
Shuiyuan300	China	*indica*	compact	H19
Zhonglouyihao 1	China	*indica*	compact	H19
Jiefangxian	China	*indica*	compact	H19
Chaoyang 1 hao	China	*indica*	compact	H19
L301B	China	*indica*	compact	H19
Baoxie 123B	China	*indica*	compact	H19
80B	China	*indica*	compact	H19

Supplementary Table S1 (continued)

Name	Origin	Species name	Phenotype	Haplotype
Baoxie-7B	China	*indica*	compact	H19
Guangluai 4 hao	China	*indica*	compact	H19
Aijiaonante	China	*indica*	compact	H19
Baigedao	China	*indica*	compact	H19
IR661-1	China	*indica*	compact	H19
Liusha 1 hao	China	*indica*	compact	H19
Erjiunan 1 hao	China	*indica*	compact	H19
Chengduai 3 hao	China	*indica*	compact	H19
Zaoshouxiangheimi	China	*indica*	compact	H19
Gongju 73	China	*indica*	compact	H19
Chikenuo	China	*indica*	compact	H19
Dongtignwanxian	China	*indica*	compact	H19
Bawangbian 1	China	*indica*	compact	H19
Xiangdao	China	*indica*	compact	H19
Xibainian	China	*indica*	compact	H19
Fanhaopi	China	*indica*	compact	H19
Xiangai B	China	*indica*	compact	H19
Zhenxian 97B	China	*indica*	compact	H19
88 B	China	*indica*	compact	H19
Gu 154	China	*indica*	compact	H19
Gui 630	China	*indica*	compact	H19
Teqingxuanhui	China	*indica*	compact	H19
Xianghui 91269	China	*indica*	compact	H19
Baoxuan 21	China	*indica*	compact	H19
Xiangwanxian 1	China	*indica*	compact	H19
Luke 3	China	*indica*	compact	H19
Aituogu 3	China	*indica*	compact	H19
Xiangwanxian 3	China	*indica*	compact	H19
Chengnongshuijing	China	*indica*	compact	H19
Jinxibai	China	*indica*	compact	H19
Xiaohonggu	China	*indica*	compact	H19
Laozaogu	China	*indica*	compact	H19
Qitougu	China	*indica*	compact	H19
Jinzhinuo	China	*indica*	compact	H19
Wuzuihonggu	China	*indica*	compact	H19
Zegu	China	*indica*	compact	H19
Minjiading 2	China	*indica*	compact	H19
Qingsiai 16B	China	*indica*	compact	H19
PeiC 112	China	*indica*	compact	H19
Yangdao 2	China	*indica*	compact	H19

Supplementary Table S1 (continued)

Name	Origin	Species name	Phenotype	Haplotype
Momi	China	*indica*	compact	H19
Aimi	China	*indica*	compact	H19
Biwusheng	China	*indica*	compact	H19
Jinyou 1	China	*indica*	compact	H19
Mowanggu	China	*indica*	compact	H19
Haoxiang	China	*indica*	compact	H19
Feienuo 2	China	*indica*	compact	H19
Xiangzaoxian 7	China	*indica*	compact	H19
Shufeng 101	China	*indica*	compact	H19
Xingguo	China	*japonica*	compact	H19
Longhuamaohulu	China	*japonica*	compact	H19
Weiguo	China	*japonica*	compact	H19
Tieganwu	China	*japonica*	compact	H19
Qitoubaigu	China	*japonica*	compact	H19
Annongwanjing B	China	*japonica*	compact	H19
Ninghui 21	China	*japonica*	compact	H19
Jan-76	China	*japonica*	compact	H19
Guihuahuang	China	*japonica*	compact	H19
Zhonghua 8 hao	China	*japonica*	compact	H19
Jindao 1hao	China	*japonica*	compact	H19
Jing 7623	China	*japonica*	compact	H19
Liaojing 287	China	*japonica*	compact	H19
Nantiangangjiugu	China	*japonica*	compact	H19
Dahongmang	China	*japonica*	compact	H19
Muxiqiu	China	*japonica*	compact	H19
Hongqi 5	China	*japonica*	compact	H19
Sanbangqishiluo	China	*japonica*	compact	H19
Mubanggu	China	*japonica*	compact	H19
Lengshuigu 2	China	*japonica*	compact	H19
Laohuzhong	China	*japonica*	compact	H19
Wuzuizi	China	*japonica*	compact	H19
Ximaxian	China	*japonica*	compact	H19
Magunuo	China	*japonica*	compact	H19
Hongkezhegu	China	*japonica*	compact	H19
Younian	China	*japonica*	compact	H19
Taichong 65	China	*japonica*	compact	H19
JWR 221	China	*japonica*	compact	H19
Lixinjing	China	*japonica*	compact	H19
Shanjiugu	China	*japonica*	compact	H19
Shoni*	Bangladesh	*indica*	compact	H19

Supplementary Table S1 (continued)

Name	Origin	Species name	Phenotype	Haplotype
Tupa 121-3*	Bangladesh	*indica*	compact	H19
Jarjan*	Bhutan	*indica*	compact	H19
Bei Khe*	Cambodia	*indica*	compact	H19
Neang Menh*	Cambodia	*indica*	compact	H19
Neang Phtong*	Cambodia	*indica*	compact	H19
Ryou Suisan Koumai*	China	*indica*	compact	H19
Jinguoyin*	China	*indica*	compact	H19
Qingyu(Seiyu) *	China	*indica*	compact	H19
Deng Pao Zhai*	China	*indica*	compact	H19
Kasalath*	India	*indica*	compact	H19
Naba*	India	*indica*	compact	H19
Puluik Arang*	India	*indica*	compact	H19
Muha*	India	*indica*	compact	H19
Jhona 2*	India	*indica*	compact	H19
Surjamukhi*	India	*indica*	compact	H19
ARC 7291*	India	*indica*	compact	H19
ARC 5955*	India	*indica*	compact	H19
Ratul*	India	*indica*	compact	H19
ARC 7047*	India	*indica*	compact	H19
ARC 11094*	India	*indica*	compact	H19
Nepal 555*	India	*indica*	compact	H19
Local Basmati*	India	*indica*	compact	H19
Padi Kuning*	India	*indica*	compact	H19
Rambhog*	India	*indica*	compact	H19
Pokkali*	India	*indica*	compact	H19
Milyang 23*	Korea	*indica*	compact	H19
Hakphaynhay*	Laos	*indica*	compact	H19
Vary Futsi*	Madagascar	*indica*	compact	H19
Shwe Nang Gyi*	Myanmar	*indica*	compact	H19
Radin Goi Sesat*	Myanmar	*indica*	compact	H19
Kemasin*	Myanmar	*indica*	compact	H19
Bingala*	Myanmar	*indica*	compact	H19
Chin Galay*	Myanmar	*indica*	compact	H19
Jene 035*	Nepal	*indica*	compact	H19
Napal 8*	Nepal	*indica*	compact	H19
Kalo Dhan*	Nepal	*indica*	compact	H19
Anjana Dhan*	Nepal	*indica*	compact	H19
Badari Dhan*	Nepal	*indica*	compact	H19
Davao 1*	Philippines	*indica*	compact	H19
IR 58*	Philippines	*indica*	compact	H19

Supplementary Table S1 (continued)

Name	Origin	Species name	Phenotype	Haplotype
Tadukan*	Philippines	*indica*	compact	H19
Calotoc*	Philippines	*indica*	compact	H19
Lebed*	Philippines	*indica*	compact	H19
Pinulupot 1*	Philippines	*indica*	compact	H19
Basilanon*	Philippines	*indica*	compact	H19
Vandaran*	Sri Lanka	*indica*	compact	H19
Bleiyo*	Thailand	*indica*	compact	H19
Tupa 729*	Bangladesh	*japonica*	compact	H19
Tima*	Bhutan	*japonica*	compact	H19
Jaguary*	Brazil	*japonica*	compact	H19
Padi Perak*	India	*japonica*	compact	H19
Phulba*	India	*japonica*	compact	H19
Nipponbare*	Japan	*japonica*	compact	H19
Khao Nok*	Laos	*japonica*	compact	H19
Khao Nam Jen*	Laos	*japonica*	compact	H19
Rexmont*	United States	*japonica*	compact	H19
Khau Mac Kho*	Vietnam	*japonica*	compact	H19
Khau Tan Chiem*	Vietnam	*japonica*	compact	H19
Urasan 1*	Japan	*japonica*	compact	H19

* , samples used to compare the nucleotide diversity.

Supplementary Table S2 Primers used in this study

Name	Type	Forward primer (5'-3')	Reverse primer (5'-3')
RM348	SSR	ccgctactaatagcagagag	ggagctttgttcttgcgaac
RM131	SSR	tcctccctcccttcgcccactg	cgatgttcgccatggctgctcc
M1	InDel	catccaaccgacacgcattttg	gatttggagcagcggatgacg
M2	SSR	cttcagcttggacaggcgatg	gaaaagagatgtctggacg
M3	InDel	cgtcgtagagctgagagctagg	ggatcagcgagctactcactcc
M4	SSR	ctgtctacaacttaacgactagc	gtggactcgctaaaactacgc
M5	SNP	gtcaaaaaaagattaagcaag	ttttatgattatgtcccacttg
M6	SNP	gcacgtgacatgagcctacacg	gcaaccttaggtcatcggttc
M7	InDel	ggaattcctacgaaaccgtggac	cagtttgacagatttagggtgc
SP1 for nucleotide diversity		ataagatggactaattggat	agattttcatgtcattgagg
SP2 for nucleotide diversity		cataaaaaggagtccagata	ttgaagtccacaaaatctcg
SP3 for nucleotide diversity		agtgaaatggagtaaaagag	agcctgccacgacaatgtag
SP4 for nucleotide diversity		ggtgcactacattactactgg	ctcaaccttacgagtcgttgtc
SP5 for nucleotide diversity		gcacgtgacatgagcctacacg	gcaaccttaggtcatcggttc
SP6 for nucleotide diversity		gggcaggctcttccacaatc	ttattgcagtgtcgctcttt
18S		gctttggtgactctagataac	gtcgggagtgggtaatttgc
OsLG1-oe for over expression[a]		ggtaccatgatgaacgttccatc	actagtgatcgaagtcgagatc
OsLG1 for In situ hybrdization		gagacggtttcgagacacagc	cgaagtcgagatcaaacatcg
*OsLG1*for quantitative RT-PCR		agcttagccttccatcagca	agatcaaacatcgcctgtcc
OsLG1 for GFP analysis[b]		atggtaccatgatgaacgttccatccgc	atgtcgacgtgatcgaagtcgagatcaaac

[a] The underline sites are *Kpn I* and *Spe I*, respectively.
[b] The underline sites are *Kpn I* and *Sal I*, respectively.

Supplementary Table S3 The cytosine sites in the promoter of *OsLG1* gene

Cyt_P	Seq_P	Cyt_P	Seq_P	Cyt_P	Seq_P	Cyt_P	Seq_P	Cyt_P	Seq_P	Cyt_P	Seq_P	Cyt_P	Seq_P
1	−5	51	−241	101	−383	151	−512	201	−693	251	−1127	301	−1238
2	−7	52	−242	102	−386	152	−523	202	−697	252	−1129	302	−1240
3	−8	53	−243	103	−388	153	−533	203	−698	253	−1135	303	−1242
4	−12	54	−247	104	−389	154	−541	204	−709	254	−1138	304	−1243
5	−51	55	−248	105	−390	155	−542	205	−710	255	−1140	305	−1245
6	−54	56	−249	106	−392	156	−544	206	−717	256	−1143	306	−1249
7	−57	57	−251	107	−394	157	−547	207	−718	257	−1144	307	−1255
8	−67	58	−252	108	−396	158	−549	208	−729	258	−1148	308	−1256
9	−68	59	−255	109	−399	159	−550	209	−732	259	−1150	309	−1258
10	−72	60	−256	110	−401	160	−553	210	−741	260	−1157	310	−1262
11	−73	61	−259	111	−404	161	−556	211	−857	261	−1159	311	−1264
12	−78	62	−260	112	−405	162	−559	212	−858	262	−1160	312	−1267
13	−79	63	−263	113	−406	163	−561	213	−860	263	−1164	313	−1270
14	−83	64	−264	114	−410	164	−562	214	−867	264	−1167	314	−1271
15	−84	65	−268	115	−411	165	−565	215	−876	265	−1170	315	−1273
16	−87	66	−270	116	−412	166	−573	216	−881	266	−1174	316	−1275
17	−89	67	−273	117	−415	167	−577	217	−886	267	−1177	317	−1279
18	−91	68	−274	118	−418	168	−857	218	−897	268	−1181	318	−1284
19	−92	69	−276	119	−420	169	−582	219	−904	269	1183	319	−1286
20	−95	70	−277	120	−421	170	−591	220	−906	270	−1184	320	−1289
21	−97	71	−278	121	−424	171	−595	221	−912	271	−1185	321	−1292
22	−98	72	−280	122	−425	172	−597	222	−913	272	−1186	322	−1293
23	−102	73	−281	123	−426	173	−599	223	−932	273	−1187	323	−1294
24	−103	74	−284	124	−428	174	−601	224	−941	274	−1190	324	−1295
25	−127	75	−286	125	−440	175	−606	225	−945	275	−1191	325	−1296
26	−131	76	−288	126	−441	176	−613	226	−959	276	1193	326	−1298
27	−133	77	−290	127	−443	177	−619	227	−962	277	−1198	327	−1301
28	−136	78	−291	128	−446	178	−620	228	−964	278	−1201	328	−1302
29	−150	79	−293	129	−447	179	−623	229	−967	279	−1204	329	−1305
30	−151	80	−295	130	−449	180	−627	230	−968	280	−1205	330	−1306
31	−152	81	−297	131	−451	181	−634	231	−969	281	−1208	331	−1307
32	−162	82	−300	132	−452	182	−641	232	−971	282	−1211	332	−1310
33	−165	83	−303	133	−453	183	−642	233	−983	283	−1215	333	−1311
34	−166	84	304	134	−454	184	−648	234	−988	284	−1218	334	−1314
35	−180	85	−308	135	−455	185	−652	235	−989	285	−1219	335	−1315
36	−183	86	−318	136	−457	186	−653	236	−990	286	−1220	336	−1327
37	−185	87	−321	137	−461	187	−657	237	−1002	287	−1221	337	−1329
38	−187	88	−324	138	−464	188	−658	238	−1003	288	−1222	338	−1332
39	−190	89	−327	139	−467	189	−659	239	−1009	289	−1223	339	−1334
40	−192	90	−331	140	−470	190	−663	240	−1012	290	−1224	340	−1342
41	−195	91	−335	141	−473	191	−664	**241**	**−1020**	291	−1225	341	−1343
42	−196	92	−341	142	−476	192	−669	242	−1050	292	−1226	342	−1347
43	−200	93	−342	143	−478	193	−674	243	−1270	293	−1227	343	−1348
44	−202	94	−351	144	−479	194	−677	**244**	**−1074**	294	−1228	344	−1352
45	−204	95	−354	145	−485	195	−678	245	−1093	295	−1229	345	−1357
46	−205	96	−357	146	−488	196	−679	246	−1096	296	−1230	346	−1358
47	−211	97	−361	147	−494	197	−680	247	−1102	297	−1231	347	−1361
48	−216	98	−362	148	−501	198	−681	248	−1117	298	−1232	348	−1362
49	217	99	363	149	−505	199	−691	249	−1121	299	−1233	349	−1364
50	−222	100	−367	150	−506	200	−692	250	−1125	300	−1235	350	−1365

Cyt_P, the cytosine position immediately upstream of the translation start site of *OsLG1* gene. Seq_P: The sequence position immediately upstream of the translation start site of *OsLG1* gene.
